# ‘Speaking Truth’ Protects Underrepresented Minorities’ Intellectual Performance and Safety in STEM

**DOI:** 10.3390/educsci7020065

**Published:** 2017-06-19

**Authors:** Avi Ben-Zeev, Yula Paluy, Katlyn L. Milless, Emily J. Goldstein, Lyndsey Wallace, Leticia Márquez-Magaña, Kirsten Bibbins-Domingo, Mica Estrada

**Affiliations:** 1 Department of Psychology, San Francisco State University, San Francisco, CA 94132, USA; 2 Department of Psychology, The Graduate Center, City University of New York, New York, NY 10017, USA; 3 Department of Biology, San Francisco State University, San Francisco, CA 94132, USA; 4 Center for Vulnerable Populations, Department of Medicine, University of California, San Francisco, CA 94143, USA; 5 Department of Social & Behavioral Sciences, University of California, San Francisco, CA 94143, USA

**Keywords:** stereotype threat, STEM, race, ethnicity, affirmation

## Abstract

We offer and test a brief psychosocial intervention, Speaking Truth to EmPower (STEP), designed to protect underrepresented minorities’ (URMs) intellectual performance and safety in science, technology, engineering, and math (STEM). STEP takes a ‘knowledge as power’ approach by: (a) providing a tutorial on stereotype threat (i.e., a social contextual phenomenon, implicated in underperformance and early exit) and (b) encouraging URMs to use lived experiences for generating be-prepared coping strategies. Participants were 670 STEM undergraduates [URMs (Black/African American and Latina/o) and non-URMs (White/European American and Asian/Asian American)]. STEP protected URMs’ abstract reasoning and class grades (adjusted for grade point average [GPA]) as well as decreased URMs’ worries about confirming ethnic/racial stereotypes. STEP’s two-pronged approach—explicating the effects of structural ‘isms’ while harnessing URMs’ existing assets—shows promise in increasing diversification and equity in STEM.

## 1. Introduction

The 2015 U.S. News/Raytheon STEM Index shows that since 2000 there has been a significant increase in employment and degrees conferred in science, technology, engineering and math (STEM) fields, but that racial/ethnic disparities remain entrenched. Black, Latina/o, and Native American individuals [henceforth referred to as underrepresented minorities (URMs)] comprise collectively only 13% of the STEM workforce and 16% of all STEM undergraduate degree recipients [1], a disconcerting statistic in a country that is predicted to become a ‘majority–minority’ nation by 2043 [2]. Sustainable racial/ethnic diversification in STEM environments requires a paradigmatic shift from the traditional ‘student-deficit’ type approach, which focuses on URMs’ lack of access to resources and lower preparedness levels, to a more comprehensive social systems approach, that explicates and addresses psychosocial barriers in order to foster STEM climates that signal intellectual safety and belonging [3,4].

Consider *stereotype threat* [5], a social contextual phenomenon that occurs when environmental signals (e.g., ‘ambient identity cues’, such as objects and art [6], and being in the numerical minority [7]) elicit worries that one’s actions will reinforce negative stereotypes about one’s race/ethnicity (e.g., being a ‘bad ambassador’) [8], eventuating in underperformance. These stereotype-based evaluative concerns have been shown to be stronger predictors for URMs’ early exit from STEM majors than lack of academic preparation [3]. The integrated process model of stereotype threat [9] offers a mechanistic explanation: evaluative concerns elicit a concomitant increase in physiological arousal and self-monitoring, resulting in reduced working memory capacity and underperformance. A chronic experience of stereotype threat is thus marked by a frequent experience of hypervigilance to evaluative situations [4,10,11].

Herein, we examine a novel and brief intervention, *Speaking Truth to EmPower* (STEP), designed to combat stereotype threat in STEM. Whereas oft-used interventions, such as values affirmation (henceforth referred to as ‘affirmation’) [12,13], sidestep informing URMs about the adverse and often implicit effects of social stereotypes, STEP takes a ‘knowledge as power’ approach, which espouses that it is both ethical and effective to be forthright with individuals about psychosocial factors that might affect them [14].

STEP consists of knowledge and actionable components. The knowledge component is a tutorial on stereotype threat, which serves to normalize and depersonalize this phenomenon by highlighting that stereotype threat is not a unique experience, but affects others as well, and by helping locate threat-induced anxiety in social systems rather than in an internal deficit [14,15]. The actionable component encourages URMs to harness their recently-acquired knowledge by capitalizing on lived experiences: to bring to mind a stereotype threat situation they experienced in the past and to then strategize about how to prepare for a similar situation in the future. The actionable component utilizes principles of active learning [16] and a ‘be prepared’ implemental-type mindset [17], documented to be efficacious for combating stereotype threat effects [18]. The actionable component is a necessary compendium piece because imparting stereotype threat knowledge by itself has been shown to be detrimental to performance [19]. Moreover, affording students the opportunity to engage with the knowledge component, by relating it to their lives, facilitates an agentic response to overcoming stereotype-based concerns. Notably, Walton and Cohen [20] have argued for and shown evidence that asking students to relate an intervention to their own lived experiences, a “saying-is-believing” effect, enables students to internalize the intervention.

The current study employs a randomized controlled trial in the College of Science and Engineering at a large California university. Participants were randomly assigned into one of the following five conditions: STEP (one of two versions: paying-it-forward or self), affirmation, in which participants selected an important value and explained how it connected them to others [13], and baseline-threat (one of two versions: standard threat or color blindness), in which no intervention was given.

The two STEP versions consisted of the same knowledge component. In the self version, participants were asked to apply the knowledge they just learned to coping with a stereotype threat situation they would likely encounter in the future. In the paying-it-forward version, participants were asked to apply it to help someone they cared about (friend, family member, or peer) cope. We suspected that the paying-it-forward version, which inherently connects one to others [13], might have been just as or even more effective than the self version. This hypothesis was exploratory and any differences, if found, were expected to showcase a matter of degree rather than a differential effect.

The two baseline-threat versions consisted of a traditional manipulation of threat [5] as well as a color blindness manipulation [21]. Given that stereotype threat research has been gaining visibility in STEM environments, we reasoned that the traditional version [5] might fail to show an effect given its possible familiarity. We thus decided to include a novel, color blindness version. Color blindness, a frequently espoused microaggression [21], denies the realities of racial/ethnic inequalities, and has been documented to have detrimental effects on URMs’ cognitive performance [22]. This hypothesis was exploratory and any differences, if found, were expected to showcase a matter of degree rather than a differential effect.

Given that the STEP and baseline-threat versions produced highly similar effects across all dependent variables (all *ps* > 0.648 and all *ps* > 0.604), we decided to aggregate them, respectively, resulting in a total of three conditions: STEP, affirmation, and baseline-threat. Participants in all conditions were placed under stereotype threat: They were given an abstract reasoning test [the Ravens Advanced Progressive Matrices (APM)] introduced as a difficult ‘puzzle’ task [23].

STEP was predicted to protect URMs’ intellectual performance, namely, URMs’ course grades and performance on the APM, because STEP makes explicit that evaluative worries are not indicative of one’s intellectual ability [14,15]. Moreover, STEP was predicted to bolster URMs’ intellectual safety, that is, URMs’ immunity to stereotype-based evaluative concerns. Herein, immunity is operationalized as a reduced preoccupation with whether one’s actions could reinforce stereotypes about one’s race/ethnicity in other people’s minds (group-reputation) and in one’s own mind (group-concept) [8]. Whereas, STEP takes stereotype threat head on, affirmation is a ‘stealth’ intervention [24], which does not afford naming and coping with stereotype-based evaluative concerns. Thus, STEP was predicted to protect URMs’ intellectual performance, on par with affirmation, and to go beyond affirmation in bolstering URMs’ intellectual safety.

## 2. Materials and Methods

### 2.1. Participants

Participants were 670 undergraduate students (414 female), enrolled in STEM majors (mathematics, engineering, physics, chemistry, biochemistry, biology, computer science, and economics) at a large California university. They completed the study online for extra credit and entry into a gift card raffle. There were 250 URMs (40 Black/African American and 210 Latina/o) and 420 non-URMs (117 White/European American and 303 Asian/Asian American). Participants’ gender and ethnicity were identified through self-report. URM and non-URM status was categorized based on guidelines outlined by the National Institutes of Health’s Diversity Statement.

### 2.2. Intervention and Dependent Measures

#### 2.2.1. STEP Intervention: Knowledge Component

Participants in STEP were first given the *knowledge component*, which consisted of a brief tutorial on stereotype threat:

We ask that you reflect on how social stereotypes can affect test performance. There are stereotypes in American society that certain groups have higher or lower intellectual ability. For example, people who are underrepresented in math and science—such as women and/or people of color and/or people of lower socioeconomic status—are often stereotyped as having inferior intellectual ability. Stereotypes can hurt performance, whether you believe in them or not.

Consider the phenomenon of stereotype threat: when a person worries about the possibility of confirming a negative stereotype about their group. How could stereotype threat affect you? If you are in a situation in which you are worried about confirming a negative stereotype about your group, you may feel additional pressure: increased stress, exaggerated preoccupation with accuracy, and decreased ability to concentrate. This additional pressure is likely to result in performance below your true ability. The good news? Knowledge is power! Stereotype threat is usually experienced without conscious awareness, but now that you know about it, you can recognize it and take steps to prevent it from affecting your performance.

#### 2.2.2. STEP Intervention: Actionable Component

Following the *knowledge component* participants received the *actionable component*, which contained a set of instructions that encouraged participants to think about a stereotype threat situation they had experienced in the past and to strategize about either helping themselves (self version) or a peer/friend (paying-it-forward version) to cope with a similar situation in the future:

In a situation where you feel anxious and out of place—for example, while taking a test or giving a presentation—you may doubt your ability to perform and/or worry about how you are being seen by others. Awareness of stereotype threat can help: recognizing and naming it when it happens helps reduce self-doubt. It is also helpful to know that these worries are normal and are felt by many people. Here is something to do: Think about a time when you might have experienced stereotype threat. Briefly describe the situation in 1–2 sentences. (If you think that stereotype threat does not affect you, please explain briefly why not.)

1. Imagine encountering a similar stereotype threat situation in the future (self version) [Imagine that someone you are close to—it can be a friend, a family member, or a peer—encounters a similar stereotype threat situation in the future (paying-it-forward version)].
2. Using your knowledge of stereotype threat, spend the next several minutes writing about what you might tell yourself in order to cope with it (self version) [ … about what you might tell this person to help them cope with it (paying-it-forward version)]. Focus on your thoughts and feelings, and do not worry about spelling, grammar, or how well written it is.

Given that there were no significant differences nor near significant differences between the *self* and paying-it-forward versions on all dependent measures (all *ps* > 0.648), they were collapsed in the analyses.

#### 2.2.3. Ravens Advanced Progressive Matrices (APM)

The APM [25], a test of abstract reasoning linked to fluid intelligence, consisted of 18 items ordered in ascending difficulty. Each item was a 3 × 3 matrix, comprised of eight visual patterns that followed a logical sequence (across and down), and a missing piece. Participants were asked to identify the missing piece from a set of possible responses. The APM has been used in stereotype threat studies as an alternative to the math-GRE [21] because it assesses logical reasoning rather than mathematical knowledge, and is thus well suited for research on students from STEM majors with varied levels of mathematical backgrounds. Given that the effects of stereotype threat are most often pronounced on the difficult portion of tasks [21], the analyses were conducted on the last six items of the APM.

#### 2.2.4. Stereotype-Based Evaluative Concerns

This measure was adapted from Shapiro’s (2011) multi-threat measure [8]. Participants were first asked to identify a negative stereotype that others might hold about their race/ethnicity, and to then write about a situation in which their actions may have been perceived by others as confirming this negative stereotype. Finally, participants were asked to complete the following measures based on what they had just written, on a 1 (not at all concerned) −7 (extremely concerned) Likert-type scale: When you are in this type of situation, to what extent are you concerned that your actions (a) will reinforce the negative stereotypes about your race/ethnicity, in other people’s minds? (group-reputation) and (b) will reinforce the negative stereotypes about your race/ethnicity, in your own mind? (group-concept) (α = 0.795).

### 2.3. Procedure

Participants were randomly assigned into one of five conditions: STEP (two versions: paying-it-forward or self), affirmation, and baseline-threat (two versions: standard threat or color blindness, see below). Given that the STEP and baseline-threat versions produced highly similar effects across all dependent variables (all *ps* > 0.648 and all *ps* > 0.604), we decided to aggregate them, respectively, resulting in a total of three conditions: STEP, affirmation, and baseline-threat.

Participants in all three conditions were placed under stereotype threat. They were given the APM, which was introduced as follows [21]: “You will now be asked to complete a puzzle-solving task. This task has been designed to be an accurate measure of your intellectual abilities, such that your score on this task predicts your success across a wide range of areas. You can expect this task to be challenging—many of the puzzle task items are difficult.” This standard manipulation of stereotype threat has been theorized to mimic the social reality of URMs in STEM, in which tests are often experienced as diagnostic of intellectual ability, and thus elicit stereotype-based evaluative concerns, underperformance and early exit [4,5].

In STEP, prior to the APM, participants were given the *knowledge component* followed by the *actionable component*. In affirmation, prior to the APM, participants were given a list of values and were then asked to pick a value that was most important to them. These values included: athletic ability, creativity, music and art, relationship with family and friends, religious values, and sense of humor. Participants were then asked to write about how this value connected them to family and friends. This version of values affirmation emphasizes social belonging, which has been shown to be the ‘active ingredient’ in values affirmation [13]. Finally, in baseline-threat, participants did not receive any intervention before taking the APM. In this condition only, half of the participants received the standard manipulation of threat (identical to the version used in STEP and affirmation see above), while the other half was given a color blindness manipulation. The latter also introduced the task as difficult but included the following statement: “ … men and women from different racial/ethnic groups have performed similarly on this task in the past.” Color blindness, a frequently espoused microaggression [21], denies the realities of racial/ethnic inequalities, and has been documented to have detrimental effects on URMs’ cognitive performance [22]. Given that there were no significant differences nor near significant differences between the standard and color blindness versions on all dependent measures (all *ps* > 0.604), they were aggregated to form a single baseline-threat condition. After participants in all three conditions completed the APM, they were given the Stereotype-Based Evaluative Concerns measure, followed by a demographics questionnaire.

### 2.4. Ethics Statement

The study and procedures were approved by the Institutional Review Board (Protocol X15–51) at San Francisco State University where data collection took place. All participants in this study provided implied consent. Participants received compensation in the form of extra course credit and entry into a gift card raffle. The data sets for this study can be found at [26].

## 3. Results

### 3.1. STEP Effects on Stereotype-Based Evaluative Concerns

**Figure 1:**
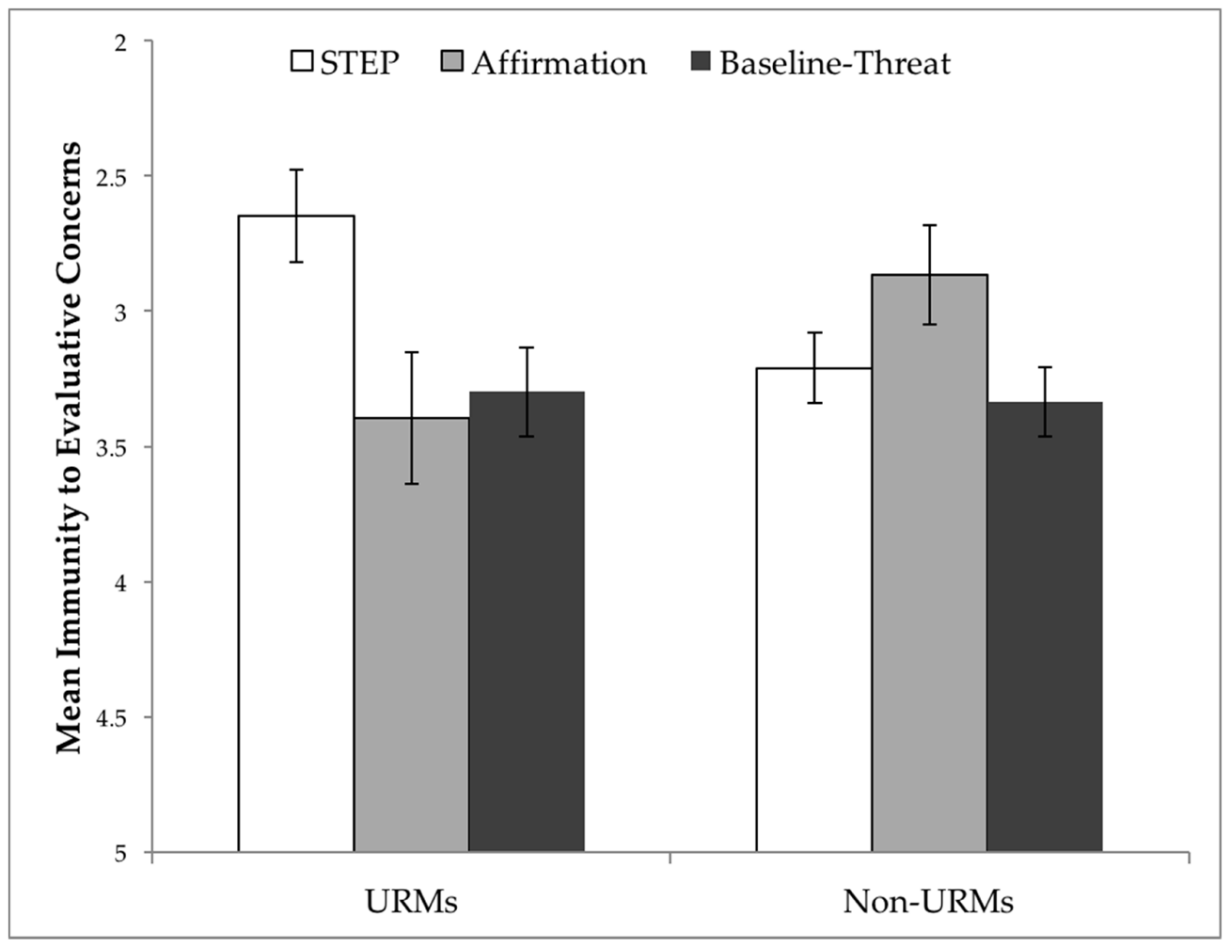
Speaking Truth to EmPower (STEP) effects on Stereotype-Based Evaluative Concerns. Mean immunity to stereotype-based evaluative concerns (a composite of group-reputation and group-concept), analyzed as a function of minority status (underrepresented minorities (URMs) vs. Non-URMs) and condition (STEP, Affirmation, Baseline-Threat). Evaluative concerns measured on a 1 (not at all concerned) to 7 (extremely concerned) Likert-type scale. The Y-axis has been inverted to visually represent immunity (i.e., decreased concerns). Error bars represent ±1 standard error. Sample distributions: URMs [STEP (*n* = 95), affirmation (*n* = 47), baseline-threat (*n* = 104)]; Non-URMs [STEP (*n* = 164), affirmation (*n* = 83), baseline-threat (*n* = 168).

A 3 × 2 between-subjects ANOVA [condition (STEP, affirmation, baseline-threat) by minority status (URMs vs. non-URMs)] on stereotype-based evaluative concerns showed a significant interaction effect, *F*(2, 655) = 4.44, *p* = 0.012, η*^2^_p_* = 0.013. As can be seen in Figure 1, there was an effect of condition, such that STEP decreased URMs’ stereotype-based evaluative concerns (i.e., a composite measure of group-reputation and group-concept), *F*(2, 243) = 4.35, *p* = 0.014, η*^2^_p_* = 0.035. Notably, URMs in STEP (*M* = 2.65, *SE* = 0.171) [but not affirmation (*M* = 3.39, *SE* = 0.243)], exhibited significantly decreased levels of evaluative concerns than URMs in baseline-threat (*M* = 3.30, *SE* = 0.164), *t*(197) = −2.63, *p* = 0.009. Non-URMs, on the other hand, did not differ significantly across conditions, *F*(2, 412) = 2.39, *p* = 0.093. This finding shows that STEP enhances URMs’ immunity to concerns about being bad ambassadors and endorsing own-group stereotypes, worries that have been shown to be central and causal to stereotype threat [5,8] and linked to early exit from STEM majors [3].

Similar interaction effects were obtained for separate analyses on group-reputation and group-concept concerns, *F*(2, 657) = 3.15, *p* = 0.043, η*^2^_p_* = 0.009 and *F*(2, 655) = 4.36, *p* = 0.013, η*^2^_p_* = 0.013, respectively. STEP decreased URMs’ group-reputation concerns (i.e., worries about confirming a negative stereotype about one’s group in other people’s minds), *F*(2, 243) = 3.89, *p* = 0.022, η*^2^_p_* = 0.031. In particular, URMs in STEP (*M* = 2.96, *SE* = 0.194) [but not affirmation (*M* = 3.81, *SE* = 0.275)], exhibited significantly decreased levels of group-reputation concerns compared to URMs in baseline-threat (*M* = 3.67, *SE* = 0.185), *t*(197) = −2.46, *p* = 0.015. In addition, STEP decreased URMs’ group-concept concerns, (i.e., worries about confirming a negative stereotype about one’s group in one’s own mind), *F*(2, 243) = 3.14, *p* = 0.045, η*^2^_p_* = 0.025. In particular, URMs in STEP (*M* = 2.34, *SE* = 0.182) [but not affirmation (*M* = 2.98, *SE* = 0.259)], exhibited significantly decreased levels of group-concept concerns compared to URMs in baseline-threat (*M* = 2.92, *SE* = 0.174), *t*(197) = −2.28, *p* = 0.024.

Of interest, the content of evaluative concerns was different for URMs and non-URMs. URMs exhibited higher frequencies of worries about STEM-related negative stereotypes regarding intellectual ability (e.g., “People think Mexicans aren’t good at sciences and math” and “Blacks don’t achieve higher education”) whereas non-URMs demonstrated more worries about privilege-related stereotypes (e.g., “ … my knowledge is taken as a function of my privilege rather than how hard I worked,” and “I make comments with good intentions that come out wrong and completely become misconstrued … [such] that I may be prejudiced”), x^2^(1) = 4.92, *p* = 0.027. This finding might explain why URMs’ and non-URMs’ levels of evaluative concerns were similar in baseline-threat.

### 3.2. STEP Effects on Abstract Reasoning

**Figure 2:**
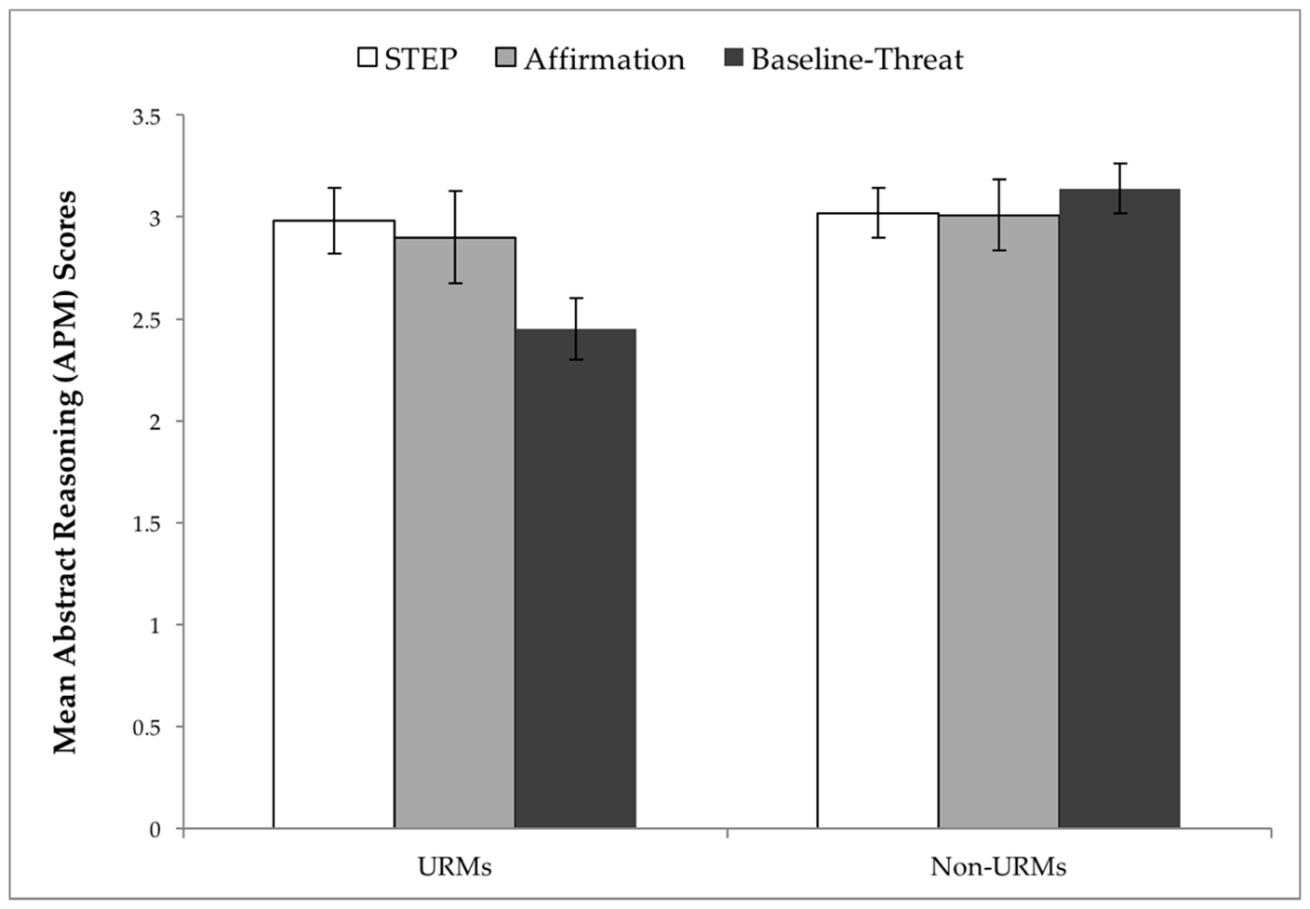
STEP effects on Abstract Reasoning. Mean abstract reasoning (APM) scores, analyzed as a function of minority status (URMs vs. Non-URMs) and condition (STEP, Affirmation, Baseline-Threat). Error bars represent ±1 standard error. Sample distributions: URMs [STEP (*n* = 95), affirmation (*n* = 48), baseline-threat (*n* = 107)]; Non-URMs [STEP (*n* = 164), affirmation (*n* = 83), baseline-threat (*n* = 168).

A 3 × 2 between-subjects ANOVA [condition (STEP, affirmation, baseline-threat) by minority status (URMs vs. non-URMs)] showed a significant interaction effect, *F*(2, 664) = 3.06, *p* = 0.047, η*^2^_p_* = 0.009 on APM scores. Specifically, there was a significant difference between URMs’ and non-URMs’ performance on the APM, such that non-URMs (*M* = 3.06, *SE* = 0.081) attained higher scores than URMs (*M* = 2.77, *SE* = 0.106), *F*(1, 664) = 4.50, *p* = 0.034, η*^2^_p_* = 0.007). Notably, STEP [similarly to affirmation (*M* = 2.90, *SE* = 0.227)] had a protective effect on URMs’ performance, such that URMs’ scores in STEP (*M* = 2.98, *SE* = 0.161) were significantly higher than their URM counterparts’ in baseline-threat (*M* = 2.45, *SE* = 0.152), *t*(200) = 2.50, *p* = 0.013, and were similar to non-URMs’ in all three conditions, all *ps* > 0.424 (See Figure 2).

Given that the effects of stereotype threat are most often pronounced on difficult tasks [21], and that the APM progresses from easy, to moderate, to difficult items, the above analyses were conducted on the last six items of the APM (a tertiary split). To examine whether problem difficulty interacted with condition and minority status, we conducted a 3 × 2 × 3 mixed-model ANOVA to test for APM performance differences across the entire set, using the number of correctly answered APM items as the dependent measure. The two between-subjects factors were condition (STEP, affirmation, baseline-threat) and minority status (URMs vs. non-URMs). The within-subjects factor was level of item difficulty [high (last six items), moderate (middle six items), low (first six items)]. This analysis revealed a condition x minority status x problem difficulty interaction *F*(4, 1306) = 2.88, *p* = 0.022, η*^2^_p_* = 0.009, such that URMs in baseline threat exhibited significantly poorer performance on the moderate (*M* = 3.53, *SE* = 0.19) and difficult items (*M* = 2.52, *SE* = 0.152) as compared to non-URMs (moderate, *M* = 4.17, *SE* = 0.15; difficult, *M* = 3.18, *SE* = 0.12), *p* = 0.009, η*^2^_p_* = 0.01, and *p* = 0.001, η*^2^_p_* = 0.018, respectively.

Overall, these findings are consistent with a classic stereotype threat effect [5], such that: (a) the only group that performed worse was the stigmatized group under threat and (b) a social contextual intervention can help level the playing field. These data provide evidence of STEP’s efficacy in enabling URMs to perform on par with non-URMs on an abstract reasoning test, introduced as a measure of intellectual ability.

### 3.3. STEP Effects on STEM Course Grades

The STEP intervention was administered online at the beginning of the Spring, 2016 semester. In contrast to the APM, which is an abstract reasoning test that does not build on previous content knowledge, course grades tend to be affected by academic preparedness. For this reason, we adjusted course grades by STEM GPA, a covariate used as a proxy for academic preparedness [27]. Course grades and STEM GPA were obtained from the Director of Institutional Research, Department of Academic Resources.

**Figure 3:**
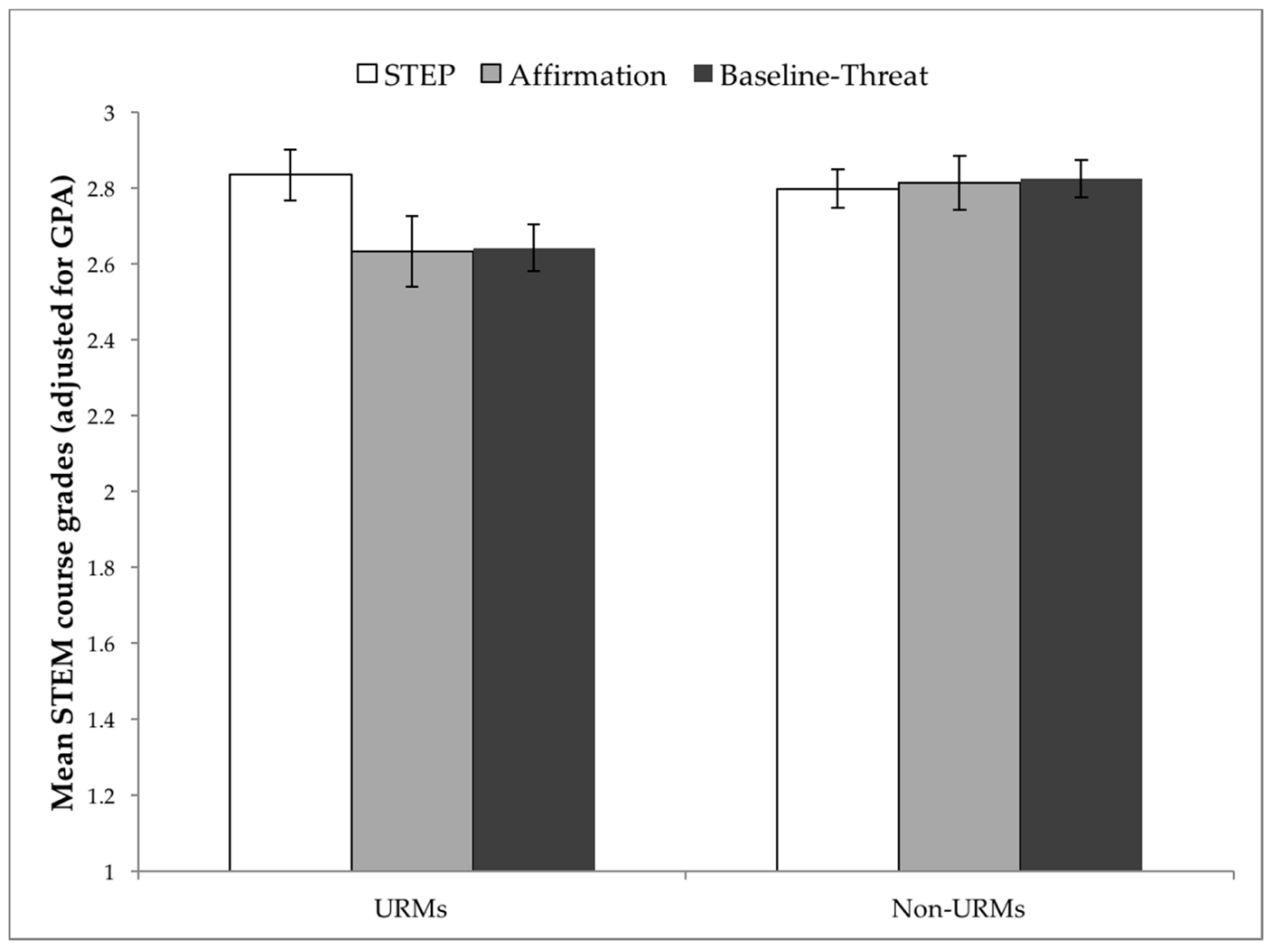
STEP effects on science, technology, engineering, and math (STEM) Course Grades. Mean STEM course grades, adjusted for STEM grade point average (GPA), analyzed as a function of minority status (URMs vs. Non-URMs) and condition (STEP, Affirmation, Baseline-Threat). The Y-axis represents GPA units ranging from 0 (=F) to 4.0 (=A). Error bars represent ±1 standard error. Sample distributions: URMs [STEP (*n* = 89), affirmation (*n* = 46), baseline-threat (*n* = 102)]; Non-URMs [STEP (*n* = 162), affirmation (*n* = 78), baseline-threat (*n* = 164).

A 3 × 2 between-subjects ANCOVA [condition (STEP, affirmation, baseline-threat) by minority status (URMs vs. non-URMs), using STEM GPA as a covariate] on course grades did not yield a statistically significant interaction effect, *F*(2, 664) = 2.09, *p* = 0.124. [Without the covariate, a 3 × 2 between-subjects ANOVA [condition (STEP, affirmation, baseline-threat) by minority status (URMs vs. non-URMs) on STEM course grades, revealed a non-significant interaction effect, *F*(2, 635) = 0.06, *p* = 0.95]. See Figure 3 for descriptives. We thus conducted simple effects to examine the relationships of interest, using a Bonferroni correction to protect against inflation of the alpha level and Type I error [28]. In baseline-threat, as predicted, there was a significant difference in STEM course grades between URMs (*M* = 2.64, *SE* = 0.063) and non-URMs (*M* = 2.83, *SE* = 0.05), *F*(1, 634) = 5.21, *p* = 0.023, η*^2^_p_* = 0.008. In contrast, in both STEP [URMs (*M* = 2.83, *SE* = 0.068); non-URMs (*M* = 2.80, *SE* = 0.05)] and affirmation [URMs (*M* = 2.63, *SE* = 0.09); non-URMs (*M* = 2.81, *SE* = 0.072], URMs and non-URMs attained similar levels of STEM course grades, *F*(1, 634) = 0.19, *p* = 0.665, η*^2^_p_* = 0.000, and *F*(1, 634) = 2.34, *p* = 0.126, η*^2^_p_* = 0.004, respectively. This pattern of results suggests that in the absence of a social contextual intervention, what may seem like a significant difference in proclivities between URMs and non-URMs might be due, in part, to the effects of an intellectually threatening environment. Thus, STEP offers some promise in closing the ethnic/racial performance gap in STEM.

With respect to all dependent outcomes (Sections 3.1–3.3), gender (while collapsing over race) did not produce significant nor near-significant interaction effects (all *ps* > 0.16). An open question is the possible intersectionality between gender and race, which lies outside the scope of the current design (and, which is underpowered for examining 12 groups). This would be an important query for future investigations.

## 4. Discussion

The phenomenon of stereotype threat has been rapidly gaining visibility in STEM environments and in popular media and thus necessitates an intervention, such as STEP, which tackles it head on [14]. STEP’s two-pronged approach—informing URMs about the adverse impact of stereotype threat, and providing opportunities for URMs to become engaged in their own intervention—not only appears to protect URMs’ intellectual performance but seems to bolster URMs’ intellectual safety, namely, immunity to stereotype-based evaluative concerns. Given the current findings, our stance is that it is both ethical and efficacious to ‘speak truth’ to URMs (and other under-represented groups) about social contextual factors that might affect them.

By elucidating how social context can elicit stereotype-based evaluative concerns, STEP enables underrepresented students to differentiate between the experience of stress and the source of that stress (located in the environment versus in the self) [14]. Attributing worries to malleable social systems rather than to fixed internal factors, allows stigmatized students to reappraise stereotype-based anxiety as naturally occurring arousal, and to use arousal as a beneficial cue for taking action that promotes agency and change [29]. STEP’s underlying theorizing aligns with that of belongingness interventions [20,30], which espouse that buffering students’ belonging uncertainty entails normalizing students’ concerns about whether they fit or are welcome in a given environment and as attenuating over time. The focus is on enabling students to change their subjective interpretation of ambiguous events (e.g., receiving critical feedback) from signaling social rejection or internal fixed deficits to being part and parcel of the normal academic experience. This kind of reappraisal helps break vicious feedback loops between students’ negative identity-based construals, stress, and impoverished academic outcomes. Whereas belongingness interventions have been designed to be “stealth,” such that their effects occur outside of conscious awareness, and to target situations that give rise to attributional ambiguity [20], STEP tackles stereotype threat head on, and thus might be especially helpful for buffering against more overt ‘isms’ in the environment. Thus, STEP is not intended to replace but, rather, to complement affirmation and belongingness interventions. The current study offers a ‘proof of concept’ that STEP helps to combat stereotype threat effects. Exact prescriptions about when and how to use STEP in conjunction with existing interventions are fodder for future research.

Non-URMs in STEP did not seem to benefit or to incur negative consequences with regard to their intellectual performance or safety. It is possible, however, that non-URMs could experience future benefits from reflecting on issues of social equity. Goff, Steele, and Davies [31] have shown that White individuals, and especially those who hold liberal views, are susceptible to stereotype threat effects in contexts in which race becomes a topic of discussion. In the current study, non-URMs expressed stereotype-based concerns about privilege, including about being perceived as racially insensitive. They were then given the opportunity to engage with these worries and have them normalized. In future investigations, we intend to conduct a longitudinal study to examine whether exposure to a “speaking truth” intervention alleviates non-URMs’ anxiety about cross-race interactions, and affects non-URMs likelihood of becoming allies, e.g., the extent of non-URMs’ involvement in campus efforts for enacting social justice change.

Like affirmation, STEP is both brief and easy to implement. An institution could adopt it for use as part of its orientation for freshmen and incoming transfer students, and/or in individual STEM courses. Regardless of the exact nature of implementation, which might differ somewhat across academic and other STEM settings, we encourage institutions to embrace reform by speaking truth that serves to empower—employing an anti-deficit rhetoric [32] for explicating the effects of structural ‘isms’ while harnessing URMs’ existing assets, resilience, and ability to turn threat into challenge.
